# Evaluation of the Becton Dickinson Rapid Influenza Diagnostic Tests in Outpatients in Germany during Seven Influenza Seasons

**DOI:** 10.1371/journal.pone.0127070

**Published:** 2015-05-26

**Authors:** Maren Eggers, Martin Enders, Elena Terletskaia-Ladwig

**Affiliations:** Prof. Gisela Enders & Kollegen MVZ GbR and the Institute of Virology, Infectious Diseases and Epidemiology e.V., Stuttgart, Rosenbergstr. 85, Stuttgart, Germany; University Hospital San Giovanni Battista di Torino, ITALY

## Abstract

**Background:**

An extensive retrospective study spanning several seasons was undertaken to evaluate the diagnostic performance of the BD rapid influenza diagnostic test (RIDT) in comparison with the RT-PCR assay.

**Methods:**

A total of 2,179 respiratory samples were tested in parallel by in-house RT-PCR and the RIDT. During the 2003-2004, 2006-2007, 2007-2008, and 2008-2009 (n=1671) seasons, the BD Directigen Flu A+B test was used, and during the 2010-2011, 2011-2012 and 2012-2013 (n=508) seasons, the BD Directigen EZ Flu A+B test b was used.

**Results:**

The sensitivity, specificity, PPV and NPV for the BD Directigen Flu A+B test calculated for types A and B together were 39%, 99%, 98%, and 56%, respectively. For the BD Directigen EZ Flu A+B test, these values were 47%, 100%, 100%, 55%, respectively. The sensitivity of the BD Directigen Flu A+B test did not differ significantly from season to season or between types A (44%) and B (37%). The sensitivity of the BD Directigen EZ Flu A+B test calculated for type A only was 59%, which was considerably higher than the sensitivity of this test for type B (23%). The sensitivity of the RIDT was approximately 40-50% in children and teenagers, but it was only 18.% in adults aged 20 years and older. The specificity of both RIDTs was very high (>99%) during all seasons.

**Conclusions:**

Due to their rapid turnaround time, RIDTs can help guide decisions about the clinical management of influenza. Because of the high specificity, a positive result can be interpreted as a true positive, and antiviral therapy as well as appropriate measures to prevent the transmission of influenza can be initiated. The best sensitivity of the RIDT is achieved in children. However, even in this group, the RIDT will only recognize influenza infection in approximately half of the cases, and influenza should still be considered in patients with negative results; negative RIDT results must be confirmed by PCR.

## Introduction

The first rapid influenza diagnostic tests (RIDTs) became available in the early 2000s [1]. These tests have the advantage of providing results within 10 to 30 minutes, and they are extremely simple to perform. The first evaluations of RIDTs were conducted using different cell culture techniques. The sensitivity of the Becton Dickinson (BD) Directigen Flu A+B test ranged from approximately 40% to 90% [2]. Subsequent studies that compared the RIDTs with more sensitive molecular techniques such as PCR found that the sensitivity of the BD Directigen Flu A+B test and the BD Directigen EZ Flu A+B test ranged from 20% to 70% [2, 3, 4, 5, 6]. Some authors have expressed disappointment with the low sensitivity of RIDTs [7]. In our laboratory, the highly sensitive RT-PCR assay is routinely used for the diagnosis of influenza. However, RT-PCR, which has a turnaround time of 4–6 h, is usually run in batches, which may delay the results. Therefore, we found RIDTs to be useful as a first-line test for specimens delivered to the laboratory in the late afternoon/evening. These specimens are immediately tested using a BD RIDT and then tested again with the more time-consuming RT-PCR assay the next morning. The results of this practice, obtained over seven influenza seasons, are analyzed in this study.

## Materials and Methods

### Samples

Respiratory specimens, which were typically nasal (cat. No. 160c rayon mini tip, Copan Diagnostics Inc., USA) and throat swabs (cat. No. 155c rayon regular tip, Copan Diagnostics Inc., USA), were obtained from pediatricians and general practitioners and were delivered to the laboratory within one or two days after collection in a transport medium containing veal infusion broth (BD Difco, USA), stabilizers, and antibiotics. Our laboratory studies have demonstrated that in this medium, the influenza virus maintains its activity for 2 days during sample transport in the winter. The majority of the specimens (90%) were obtained from the south of Germany and originated from outpatients suspected of having an influenza infection.

A total of 2,179 samples were tested in parallel for the influenza virus using an in-house RT-PCR assay and the BD RIDT during seven different influenza seasons: 2003–2004, 2006–2007, 2007–2008, 2008–2009, 2010–2011, 2011–2012, and 2012–2013. During the pandemic influenza season spanning from August 2009 until March 2010, the diagnosis of influenza was performed using RT-PCR only. Therefore, data from this season were not included in the evaluation of the BD RIDT.

The samples used in this study were taken from patients in all age groups. An analysis of the age distribution of influenza infection was performed using a total of 17,626 samples, which were tested by RT-PCR during the 2006–2007, 2007–2008, 2008–2009, 2009–2010 (pandemic), 2010–2011, 2011–2012 and 2012–2013 seasons.

### Ethics Statement

Ethical approval was not required for this study because all samples were collected for routine laboratory analysis of influenza virus infection and were tested anonymously. The study was carried out in compliance with the Helsinki Declaration.

### RIDT

During the 2003–2004, 2006–2007, 2007–2008, and 2008–2009 seasons, the BD Directigen Flu A+B test was used, and during the 2010–2011, 2011–2012 and 2012–2013 seasons, the BD Directigen EZ Flu A+B test was used. The samples were mixed in the transport medium for 1 min using a vortex, and the assays were performed according to the manufacturer’s instructions.

### RT-PCR

#### Automated nucleic acid isolation

Viral RNA preparation was performed on an automated MagNA pure instrument using the MagNA Pure LC total nucleic acid isolation kit (Roche Diagnostics GmbH, Mannheim, Germany). Briefly, the input sample volume was 200 μl, and the nucleic acids were eluted in a final elution volume of 50 μl.

#### Real-time reverse transcription-PCR

Real-time RT-PCR was done using the LightCycler system. Amplification was performed in a 20 μL reaction volume consisting of 10 μL of kit-supplied QuantiTect Probe Master Mix (QIAGEN, Hilden, Germany), 0.5 μM of each primer, 0.18 μM probe, 0.2 μL kit-supplied QuantiTect RT mix, and 6.8 μL of purified RNA. Primers and probes were described previously [8, 9]. Real-time PCR was carried out with an initial RT step at 50°C for 20 min, followed by PCR activation at 95°C for 15 min and 50 cycles of amplification (95°C for 5 s, 55°C for 20 s, 72°C for 30 s). Fluorescence development was measured once each cycle after the elongation step.

#### Typing of viruses

The RT-PCR assay was able to differentiate between type A and type B influenza. Further characterization was performed after isolation of the virus in cell culture. During the 2003–2004 to 2007–2008 seasons, the virus was isolated using Madin-Darby canine kidney (MDCK) cells by conventional cell culture, rapid cell culture and MDCK 33016PF suspension culture, which have been described previously [10, 11]. During the 2008–2009 and 2010–2011 seasons, PCR-positive samples were inoculated onto 24-well tissue culture plates with MDCK and HepG2 cells (liver hepatocellular cells). The plates were centrifuged at 1200×g for 30 min. The supernatant was then removed, and 1 ml of Eagle’s Minimal Essential Medium (EMEM) containing TPCK trypsin (2 μg/ml) and antibiotics was added. The plates were incubated in a humidified chamber at 37°C for 6 days under 5% CO_2_. Influenza infection in the cell cultures was confirmed by staining the cells with monoclonal antibodies against influenza A or B (Chemicon/Millipore, Temecula, CA) as described for rapid or conventional cell culture. Positive supernatants were sent to the Robert Koch Institute (RKI, National Reference Centre) and were typed using the hemagglutination inhibition (HI) assay with RKI and WHO pools of specific antisera. The HI procedure for subtyping influenza virus isolates has been described previously [11, 12]. Briefly, specific antisera raised in ferrets were treated with a receptor-destroying enzyme. The HI assays were performed using 4 hemagglutination units of virus and 0.75% (vol/vol) guinea pig erythrocytes.

### Statistical analyses

The accuracy of the BD RIDT was estimated in comparison with RT-PCR as a reference test. The sensitivity, specificity, and positive and negative predictive values were calculated using two-by-two contingency tables. The 95% confidence intervals (CI) for these parameter were calculated using Wilson’s efficiency score. Comparisons of the sensitivities of the assays for the A and B types, as well for the different seasons and various age groups, were performed using the chi-squared test.

## Results

The accuracy of the BD Directigen Flu A+B influenza rapid test was determined in 1,671 samples, and the accuracy of the BD Directigen EZ Flu A+B test was determined in 508 samples by calculating the sensitivity and specificity and the positive and negative predictive values using RT-PCR as a gold standard. The results are presented in Table 1. The sensitivity of the BD Directigen Flu A+B test did not differ significantly from season to season (P = 0.381), and it varied from 49% during the 2003–2004 season to 36% during the 2008–2009 season. The average sensitivity of the BD Directigen Flu A+B test was 39%. The sensitivity of the BD Directigen EZ Flu A+B test was 47%, which was slightly but not significantly higher than that of the BD Directigen Flu A+B test (P = 0.104) (Table 1).

**Table 1:** Accuracy of the BD Directigen Flu A+B test and the BD Directigen EZ Flu A+B test during different influenza seasons calculated using the RT-PCR (A+B) assay as a gold standard.

	PCR negative	PCR positive	Sensitivity % (95%CI)	Specificity % (95% CI)	PPV % (95% CI)	NPV % (95% CI)
**BD Directigen Flu A+B (RT)**						
**2003–2004**						
negative	172	23	49 (34–64)	100 (97–100)	100 (81–100)	88 (82–92)
positive	0	22				
n = 217	172	45				
**2006–2007**						
negative	212	155	40 (34–46)	99 (97–99)	99 (94–99)	57 (52–62)
positive	1	105				
n = 473	213	260				
**2007–2008**						
negative	126	222	38 (33–43)	97 (92–99)	97 (92–99)	36 (31–41)
positive	4	136				
n = 488	130	358				
**2008–2009**						
negative	231	166	36 (30–42)	99 (96–99)	97 (91–99)	58 (53–63)
positive	2	94				
n = 493	233	260				
**Total**						
negative	741	566	39 (35–42)	99 (97–99)	98 (95–99)	56 (53–59)
positive	7	357				
n = 1671	748	923				
**BD Directigen EZ Flu A+B (RT)**						
**2010–2011**						
negative	56	42	51 (40–62)	100 (92–100)	100 (90–100)	57 (47–67)
positive	0	45				
n = 143	56	87				
**2011–2012**						
negative	57	37	41 (29–54)	100 (92–100)	100 (83–100)	60 (49–70)
positive	0	26				
n = 120	57	63				
**2012–2013**						
negative	87	83	47 (39–55)	98 (91–99)	98 (91–99)	51 (43–58)
n positive	1	74				
n = 245	88	157				
**Total**						
negative	200	162	47 (41–53)	100 (97–100)	99 (95–99)	55 (50–60)
positive	1	145				
n = 508	201	307				

Key: CI—confidence interval

PPV—positive predictive value

NPV—negative predictive value

The specificities of both BD RIDTs were very high for all seasons. The specificities of the rapid tests, which are shown in Table 1, were calculated in comparison to RT-PCR. During the 2007–2008 season, the influenza A virus was isolated from two samples in cell culture that were found to be negative by RT-PCR but positive by the rapid test. Accordingly, of a total of 949 RT-PCR-negative samples, only 6 were false positives according to the rapid test. Thus, the specificity of the rapid test was nearly 100%.

**Fig 1:**
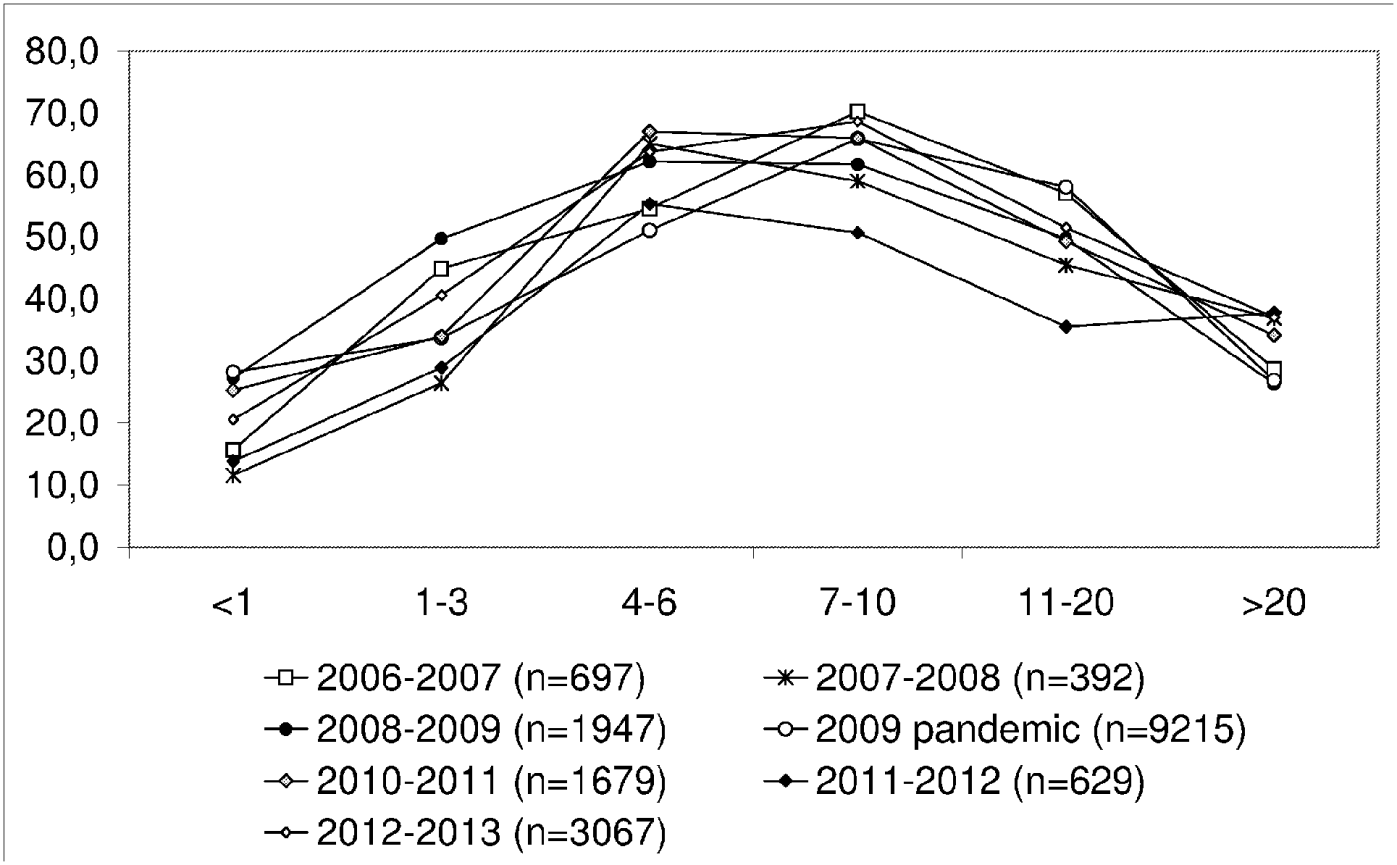
Percentage of RT-PCR-positive samples from various age groups and during various seasons.

Over the course of the study, different strains of influenza were isolated (Table 2). The sensitivity of the BD Directigen Flu A+B test for type A strains was found to be slightly, but not significantly, higher than for the sensitivity for type B strains (P = 0.124) (Table 3). The sensitivity of the BD Directigen EZ Flu A+B test was considerably higher for type A influenza (P = 0.007) (Table 4). The specificity of both RIDTs for type A and type B influenza and the corresponding positive predictive values were nearly 100%. The negative predictive value of the BD Directigen Flu A+B test was 80 (78–81)% for influenza A and 87 (85–89)% for influenza B. The negative predictive value of the BD Directigen EZ Flu A+B test was 79 (74–82)% and 83 (79–86)%, respectively. The respiratory samples used in this study were collected from patients in all age groups. Analysis of the age distribution of influenza infections was performed using a total of 17,626 samples, which were tested by RT-PCR during the 2006–2007, 2007–2008, 2008–2009, 2009–2010 (pandemic), 2010–2011, 2011–2012, and 2012–2013 seasons. The positive detection rate was the highest in the group of kindergarten and school-aged children (Fig 1). The same age distribution was observed in the samples used for comparison of the BD Directigen Flu A+B test with the RT-PCR assay. Furthermore, Table 5 illustrates that the sensitivity of the rapid test in children and teenagers in comparison to RT-PCR was significantly higher than in adults over 20 years of age (P = <0.001). Half of the toddlers and children that tested positive by PCR also tested positive with the BD rapid test. However, only one in five adults positive by PCR was also positive with the BD rapid test.

**Table 2:** Results of viral Typing in PCR positive samples.

Season	A total	A strains subtyped	A+B	B total	B strains subtyped	Total
**2003–2004**	45		-	-	-	45
**2006–2007**	207	21 x A/New Caledonia/20/99 (H1N1)-like		5	5 x B/Malaysia/256/04-like	212
		109 x A/Wisconsin/67/05 (H3N2)-like				
		1 x A/California/07/04 (H3N2)-like				
**2007–2008**	202	2 x A/Brisbane/59/07 (H1N1)-like	8	150	1 x B/Jiangsu/10/03-like	360
		3 x A/Fukushima/141/06 (H1N1)-like			45 x B/Florida/04/06-like	
		49 x A/Solomon Islands/3/06 (H1N1)-like				
**2008–2009**	176	3 x A/Brisbane/59/07 (H1N1)-like	12	72	1 x B/Florida/04/06-like	260
		93 x A/Brisbane/10/07 (H3N2)-like			58 x B/Victoria/02/87-like	
**2010–2011**	73	38 x A/California/07/09 (H1N1)-like		14	1 x B/Florida/04/06-like	87
					13 x B/Brisbane/60/08-like	
**2011–2012**	46	2 x A/California/07/09 (H1N1)-like		20	8 x B/Brisbane/60/08-like	66
		2 x A/Iowa/19/2010 (H3N2)-like			7 x B/Bangladesh/3333/07-like	
		29 x A/Perth/16/09 (H3N2)-like			2 x B/Florida/4/06-like	
		2 x A/Stockholm/18/2011 (H3N2)-like				
**2012–2013**	81	15 x A/California/07/09 (H1N1)-like	4	72	5 x B/Brisbane/60/08-like	157
		12 x A/St.Petersburg/27/2011 (H1N1)-like			1 x B/Wisconsin/1/2010-like	
		9 x A/Victoria/361/2011 (H3N2)-like			17 x B/Estonia/2011-like	
**total**	**830**		**24**	**333**		**1187**

**Table 3:** The accuracy of the BD Directigen Flu A+B influenza rapid test vs. RT-PCR for A and B.

BD Directigen Flu A+B (RT) (Seasons 2003–2004, 2006–2007, 2007–2008 and 2008–2009)	PCR negative	PCR positive	Sensitivity % (95%CI^1^)	Specificity % (95% CI)	PPV^2^% (95% CI)	NPV^3^% (95% CI)
**A**						
negative	1356	346	44 (40–48)	100 (99–100)	98 (97–100)	80 (78–81)
positive	3	267				
n = 1972	1359	613				
B						
negative	968	141	37 (31–44)	100 (99–100)	97 (90–99)	87 (85–89)
positive	3	84				
n = 1196	971	225				

Key: CI—confidence interval

PPV—positive predictive value

NPV—negative predictive value

**Table 4:** The accuracy of the BD Directigen EZ Flu A+B influenza rapid test vs. RT-PCR for A and B.

BD Directigen EZ Flu A+B (RT (Seasons 2010–2011, 2011–2012 and 2012–2013)	PCR negative	PCR positive	Sensitivity % (95%CI)	Specificity % (95% CI)	PPV % (95% CI)	NPV % (95% CI)
**A**						
negative	303	82	59 (52–66)	100 (98–100)	99 (95–100)	79 (74–82)
positive	1	118				
n = 504	304	200				
B						
negative	398	82	23 (15–31)	100 (99–100)	100 (83–100)	83 (79–86)
positive	0	24				
n = 504	398	106				

Key: CI—confidence interval

PPV—positive predictive value

NPV—negative predictive value

**Table 5:** Accuracy of BD rapid influenza diagnostic tests (RIDTs) in different age groups.

BD Directigen Flu A+B test and BD Directigen EZ Flu A+B test	PCR negative	PCR positive	Prevalence % (95%CI)	Sensitivity % (95% CI)	Specificity % (95% CI)	PPV (%)	NPV (%)
**<1**							
negative	49	16	38(27–50)	47(29–65)	100(91–100)	100(73–100)	75(63–85)
positive	0	14					
n = 65							
**1–3**							
negative	232	120	49(44–53)	46(40–53)	99(96–100)	97(91–99)	66(61–71)
positive	3	104					
n = 352							
**4–6**							
negative	151	140	64(60–68)	48(42–54)	99(96–100)	99(95–100)	51(46–58)
positive	1	128					
n = 291							
**7–10**							
negative	126	173	70(66–75)	43(37–49)	98(94–100)	98(94–100)	42(37–48)
positive	2	130					
n = 299							
**11–20**							
negative	185	133	55(50–60)	42(36–48)	100(97–100)	100(94–100)	58(52–64)
positive	0	97					
n = 318							
**>20**							
negative	174	134	48(43–54)	18(12–25)	99(94–99)	97(81–100)	57(51–62)
positive	1	29					
n = 308							
**Total**							
negative	917	716	57(55–59)	41(38–44)	99(98–100)	98(97–99)	56(54–59)
positive	7	502					
n = 1633	925	1218					

Key: CI—confidence interval

PPV—positive predictive value

NPV—negative predictive value

## Discussion

RIDTs detect the influenza antigen using monoclonal antibodies. Monoclonal antibodies against a conserved nucleoprotein are commonly used to detect all strains and subtypes of the influenza virus. The findings of several evaluation reports demonstrate that various RIDTs, such as the Inverness Medical BinaxNOW Influenza A&B test (Binax, Inc., Scarborough, Maine); the BD Directigen EZ Flu A+B test (Becton, Dickinson and Company, Sparks, Maryland); and the Quidel QuickVue Influenza A+B test (Quidel Corporation, San Diego, California), have comparable sensitivities and specificities [4, 13, 14, 15]. Over the last decade the RIDTs have been modified and improved by their manufacturers. Initially, BD Diagnostics provided the BD Directigen Flu A+B test, which is based on enzyme immunoassay technology, and later, the BD Directigen EZ Flu A+B test, which is based on lateral flow technology. According to the BD package insert for the BD Directigen Flu A+B test, viral particles present in the specimen bind non-specifically to the membrane. After washing, these particles are visualized using conjugated anti-influenza antibodies. In the EZ test, anti-influenza antibodies are printed on the membrane. In brief, the influenza A or B viral antigens present in a respiratory specimen bind to the anti-influenza antibodies conjugated to visible molecules on the corresponding A and B test strips. The antigen-conjugate complex migrates across the test strip to the reaction area and is captured by the line of antibody on the membrane. For improvement of antigen presentation, the BD Directigen EZ Flu A+B test utilizes a reagent that breaks down mucoid specimens. In 2011, the most recent improved version of the BD RIDT was issued. The new BD Veritor System incorporates a digital reader for the evaluation and interpretation of results and is included in the Clinical Laboratory Improvement Amendments (CLIA) Waived Test List. In a small study, the BD Veritor was ten times more sensitive than the Directigen EZ Flu A+B in tests for detecting influenza strains propagated in cell culture [16]. This innovation improves the accuracy of the test evaluation and eliminates the need for subjective interpretation, but it remains unknown whether the sensitivity of the test for clinical specimens is improved. Clinical studies are currently being conducted to investigate the sensitivity of the BD Veritor in clinical samples.

The sensitivity of the investigated RIDTs for types A and B influenza taken together ranged approximately from 40% to 50%. The sensitivity of the BD Directigen EZ Flu A+B test for the more pathogenic and common influenza A strains, calculated independently from type B influenza, was 54%. As demonstrated in Table 6, this result is comparable to the results of other studies [2, 3, 4, 5, 6, 17]. This means that approximately half of the cases of influenza would be diagnosed. In contrast to the low sensitivity, the test has been shown to have a high specificity, greater than 99%. False-positive results were rare, and accordingly, the PPV was high. Thus, a positive result confirms that the patient has influenza, but a negative result does not exclude influenza.

**Table 6:** Sensitivity of BD RIDTs for Influenza A in comparison to RT-PCR reported in different studies.

Reference	Region	season	Test	total number of samples (n)	No. of Influenza A PCR positive samples (n)	Sensitivity for Influenza A (%)	Specificity for Influenza A (%)	PPV (%)	NPV (%)
This study	Germany	2003–2004	BD Directigen Flu A+B		267	44	44		
		2006–2007							
		2007–2008							
		2008–2009							
		2010–2011	BD Directigen EZ Flu A+B		200	59	59		
		2011–2012							
		2012–2013							
Al Johani et al., 2011 [5]	Saudi Arabia	June 2009	BD Directigen EZ Flu A+B	143	34	21	99	87.5	80.0
			TruFlu	143	5	10	98	60.0	77.5
Cheng et al., 2011 [6]	China	September 2009	BD Directigen EZ Flu A+B	807	235	71	99.8	99.4	89.6
			FluA Dot	807	235	91	99.7	99.1	97.0
Blázquez et al., 2010 [17]	Spanien	Juni-August 2009	BD Directigen EZ Flu A+B		71	70	100	100	76.6
Vasoo et al., 2009 [4]	USA	May- June 2009	BD Directigen EZ Flu A+B	n.d.	84	47	100	100	89.6
			BinaxNOW Influenza A&B	n.d.	84	38	100	100	88.2
			QuickVue Influenza A+B	n.d.	84	53	100	100	90.8
Liao et al., 2009 [2]	Canada	2006–2007	BD Directigen Flu A+B	180	51	59	99.2	96.8	85.9
		2007–2008							
Gröndahl et al.,2005 [3]	Germany	2002–2003	BD Directigen Flu A+B	299	41	29	99.2	85.7	89.9

n.d. not determined

Different factors that may influence the sensitivity of the RIDTs were investigated in this study. Considering that the monoclonal antibodies used in the RIDTs are directed against a conserved nucleoprotein, antigenic differences between type A and type B influenza should not affect the sensitivity. However, the BD Directigen EZ Flu A+B test had a lower sensitivity for influenza B strains than for influenza A strains. A lower sensitivity for the B strains was also shown in other studies [2, 3]. In contrast, as shown in Table 1, variations in the circulating influenza A viruses from season to season e.g., an H1N1 season or an H3N2 season, do not interfere with the sensitivity of the RIDTs.

However, the sensitivity of the RIDT was strongly dependent on the patient’s age. In this study, the test demonstrated very low sensitivity in adult patients. In the group of patients over 20 years old, the sensitivity of the RIDT was only 18.0%. This may be explained by the low excretion of the influenza virus in adults. This strong age dependence of viral shedding after vaccination with a live FluMist vaccine was observed in a clinical trial (NCT00192140) conducted in healthy individuals ranging from 6 months to 49 years in age [18]. The virus titer determined using the nasal secretions of adults was 100 times less than that of children under two years of age [19]. Moreover, the adults become infected with influenza more rarely. The highest proportion (89%) of subjects shedding the virus was found in children under 23 months, and only 20% of adults over 18 years old were found to shed virus [19]. Also in our study the influenza positive-detection rate was highest for the group of kindergarteners and school-aged children and lowest for adults, for both, the RT-PCR assay and the RIDT (Fig 1 and Table 5). PPV and NPV of a test are influenced by the prevalence. For that reason, as well, it is more likely that school children, who were tested positive, have influenza. For adults, the sensitivity of the test is very low, however the lower prevalence of influenza infection in this group makes it more likely, that adults who were tested negative, are truly negative. Equally, as illustrates by the NPV, not all negative results will represent no disease.

Additionally, the sensitivity of the RIDT depends on the time between the onset of symptoms and sample collection. Recently, it was shown that testing too early (11 h after the onset of symptoms) can increase likeliness of a false-negative result [20, 21]. During the peak of infection, 36 to 72 hours after onset, the sensitivity of the test increased, but in that moment approximately 30 to 50% of the epithelial cells in the upper respiratory tract are destroyed [22] and it can be too late for antiviral therapy. Therefore, for optimal application of the RIDT to support antiviral therapy, the replication kinetics of influenza must be considered.

Despite the confusion about the new systematic review of neuraminidase inhibitors (oseltamivir, zanamivir) for influenza in the Cochrane Library and the British Medical Journal [23, 24, 25], the CDC and other societies such as the Infectious Diseases Society of America (IDSA) continue to recommend the use of neuraminidase inhibitors for the treatment of influenza as soon as possible for patients who are severely ill and for those who are at the greatest risk for complications from influenza. [26]. The CDC states that this recommendation is supported by a recent meta-analysis of 29,000 patients who were hospitalized due to infection with the 2009 H1N1 influenza virus during the 2009–10 pandemic [27]. Muthuri et al. showed in their study that neuraminidase inhibitor treatment of adults (> 16years) have a 25% reduced mortality risk, and additionally, early treatment was associated with a 50% reduction risk of death compared with no treatment. The study confirms that patients with early antiviral treatment within 48 hours after illness onset are the most likely to benefit [27, 28]. Furthermore, preventive treatment with antiviral drugs leads to a reduction of transmission rates [29].

Taken together, because of their fast turnaround time and despite their low sensitivity, RIDTs can be useful for the rapid diagnosis of influenza during the epidemic season. The use of these tests may guide decisions regarding when to begin antiviral therapy and implement preventive measures against spread of the infection as early as possible. For the clinician, this means that although false negatives are frequent, a positive result should be interpreted as a true positive. The highest sensitivity of the RIDT can be achieved in children and teenagers from one to three days after the onset of the disease. The possibility of false-negative results is high in samples taken immediately after disease onset and in samples obtained from adults. A negative RIDT result must be confirmed by RT-PCR if the result is likely to affect patient management. [14].
